# Items

**Published:** 1875-04

**Authors:** 


HIGH TEMPERATURE. A report was recently presented to the Clinical Society of London, of a case in which an extraordinarily high temperature was developed. The patient, a young lady, was thrown from a horse and sustained very severe injuries, during the subsequent illness the temperature reached at one time 122°, and for two weeks was never below 108°. The patient recovered. The observations were confirmed by different physicians, and the employment of a number of thermometers.——NEW MEDICAL SOCIETY IN BUFFALO. We understand that a meeting of Physicians has been called for the purpose of forming a new Medical Society in this city, to be termed we believe, A Clinical Society. We wish the projectors all success.——A STEP IN THE RIGHT DIRECTION. The authorities of the Medical School of Harvard College announce that on and after September 1877 all students seeking admission to the Medical School must present a degree in Letters, or Science, from a recognized College or Scientific School, or pass an examination in, 1. Latin. The translation of easy Latin prose. French or German will be accepted as a substitute. 2. Physics. Candidates will be expected to show such a knowledge of this subject as may be obtained from Balfour Stewart's Elementary work on Physics.——SALICYLIC ACID sells in New York at eighty cents per ounce.——NEW YORK ACADEMY OF MEDICINE. The amount paid toward the building fund of the New York Academy of Medicine has reached $32,500. With but few exceptions the money has been paid by members of the Academy.——HOLLOWAY'S ASYLUM. The following inscription is proposed by *Punch* for the front of the idiot asylum, founded by Mr. Holloway, whose fortune was made by the sale of Patent Medicines:

"How oft is fate so just: see wealth restored,
Back to the simple source, from whence it poured."

CAUSE OF INVERTED NIPPLES. A writer in the *Medical Investigator*, (Homœopathic), recommends *Apis* for inverted nipples. The explanation of the action of the remedy is that it has great power over ovarian difficulties, and that the patient is hysterical and "shrinks when the baby touches the nipple, or at the thought of its doing so, and thus by the *jumping of the ovary* the nipple is drawn in." If that explanation can be beaten we should be glad to hear of it.——CHINCO-QUININE. The reliability of this drug being questioned. Messrs. Clapp & Co. have procured the following analyses:

*Chemical Laboratory of University of Pennsylvania.*

WEST PHILADELPHIA, JANUARY 29, 1875.

MESSRS. BILLINGS, CLAPP & CO.

*Gentlemen*—I have received by express a package marked, "sealed by S. P. Sharples, January 22, 1875," and containing a bottle of Cincho-Quinine, with the label of James R. Nichols & Co., Chemists, Boston, which I have tested, and have found it to contain *quinine*, *quinidine*, *cinchonine* and *cinchonidine*.

Yours Respectfully, F. A. GENTH,
Professor of Chemistry and Mineralogy.

---

*Laboratory of the University of Chicago.*

CHICAGO, FEBRUARY 1, 1875.

I hereby certify that I have made a chemical examination of the contents of a bottle of Cincho-Quinine, and by direction I made a qualitative examination for *quinine*, *quinidine* and *cinchonine*, and hereby certify that I found these alkaloids in Cincho-Quinine.

C. GILBERT WHEELER,
Professor of Chemistry

